# P05.28. Current practice amongst acupuncturists treating threatened miscarriage in Australia and New Zealand

**DOI:** 10.1186/1472-6882-12-S1-P388

**Published:** 2012-06-12

**Authors:** D Betts, C Smith, H Dahlen

**Affiliations:** 1 University of Western Sydney, Penrith South, NSW, Australia

## Purpose

To investigate current use and clinical practice considerations amongst acupuncturists treating threatened miscarriage, a common complication of pregnancy that may result in miscarriage and premature labour. Although only a “wait and see approach” is advised medically, treatment recommendations exist within acupuncture texts. However these are conflicting, potentially creating treatment uncertainty and limiting clinical practice. As women increasingly seek acupuncture for their fertility, opportunities exist to offer interested women treatment for threatened miscarriage. To explore this potential, acupuncturist’ views were sought to add to the limited information currently available.

## Methods

A mixed methods study involving a self-completed questionnaire and semi-structured interviews was utilized. An online survey link was sent through Australia and New Zealand acupuncture associations requesting practitioners’ views on safety concerns and specific treatment modalities. Descriptive statistics were used to analyse data. Thirteen participants were purposefully selected for interviews to further explore perceptions of clinical practice. Interviews were conducted and recorded via Skype, transcribed verbatim and analysed through thematic analysis.

## Results

Of the 370 respondents, 214 (58%) had treated women for threatened miscarriage within the previous year. Detailed responses about current practice were obtained from 164 practitioners. Their safety concerns focused on inexperienced practitioners causing miscarriage. Only 16% saw Western medical information as useful. While the majority avoided points traditionally used to induce labour, 13% would use LI 4, 22% SP 6 and 38% BL 31. Clinical practice reflected the diverse treatment strategies within acupuncture texts. Interviews illustrated how practitioners integrated this diversity with themes including ‘rational practice,’ justifying the use of contraindicated points through theory and personal experience, ‘reflective learning’ and ‘treatment responsibilities - more than needles’.

## Conclusion

Practitioners demonstrated an interest in treating threatened miscarriage and responded positively to clinical practice questions. Feedback gathered contributes to informing clinical practice for this common complication of pregnancy.

